# Repression of TGF-β Signaling in Breast Cancer Cells by miR-*302/367* Cluster

**DOI:** 10.22074/cellj.2020.6193

**Published:** 2019-07-31

**Authors:** Mona Ahmadalizadeh Khanehsar, Moslem Hoseinbeyki, Masoumeh Fakhr Taha, Arash Javeri

**Affiliations:** 1 Department of Stem Cells and Regenerative Medicine, Institute for Medical Biotechnology, National Institute of Genetic Engineering and Biotechnology (NIGEB), Tehran, Iran; 2 Department of Biology, Damghan Branch, Islamic Azad University, Damghan, Iran

**Keywords:** Breast Cancer, miR-302/367, Reprogramming, Transforming Growth Factor-Beta

## Abstract

**Objective:**

Epigenetic alterations of the malignantly transformed cells have increasingly been regarded as an important event in the carcinogenic development. Induction of some miRNAs such as miR-302/367 cluster has been shown to induce reprogramming of breast cancer cells and exert a tumor suppressive role by induction of mesenchymal to epithelial transition, apoptosis and a lower proliferation rate. Here, we aimed to investigate the impact of miR-302/367 overexpression on transforming growth factor-beta (TGF-β) signaling and how this may contribute to tumor suppressive effects of miR-302/367 cluster.

**Materials and Methods:**

In this experimental study, MDA-MB-231 and SK-BR-3 breast cancer cells were cultured and transfected with miR-302/367 expressing lentivector. The impact of miR-302/367 overexpression on several mediators of TGF-β signaling and cell cycle was assessed by quantitative real-time polymerase chain reaction (qPCR) and flow cytometry.

**Results:**

Ectopic expression of miR-302/367 cluster downregulated expression of some downstream elements of TGF-β pathway in MDA-MB-231 and SK-BR-3 breast cancer cell lines. Overexpression of *miR-302/367* cluster inhibited proliferation of the breast cancer cells by suppressing the S-phase of cell cycle which was in accordance with inhibition of TGF-β pathway.

**Conclusion:**

TGF-β signaling is one of the key pathways in tumor progression and a general suppression of TGF-β mediators by the pleiotropically acting miR-302/367 cluster may be one of the important reasons for its anti-tumor effects in breast cancer cells.

## Introduction

Despite recent advancements in the treatment of breast cancer, it still remains one of the leading causes of cancer deaths among women (1). Therefore, development of new therapeutic approaches for breast cancer is of the utmost importance. Reprograming of somatic cells and generation of induced pluripotent stem (iPS) cells by some transcription factors including OCT3/4, SOX2, NANOG, KLF4, LIN28 and MYC (2, 3) demonstrated that the cell fate can be manipulated *in vitro*. Reprogramming is a process accompanied by distinct alterations in chromatin and transcriptional programs. 

MiRNAs constitute a class of 17-24 bp small non-coding RNAs, involved in regulation of different biological processes and cancer-related cellular activities such as apoptosis, proliferation and invasion (4, 5). MiR-302/367 cluster possesses a coding sequence located in intron 8 of the *LARP7* gene and codes for 5 miRNAs including miR302a, miR302b, miR302c, miR302d, and miR367 which are highly expressed in embryonic stem cells (6-8), but their expression decline rapidly after differentiation (9). It was shown that miR-302/367 cluster can effectively reprogram human and mouse somatic cells to iPS cells (10, 11). miR-302 is also able to reprogram human cancer cells to a human embryonic stem cell-like state with a slow cell cycle rate and dormant cell-like morphology (12, 13). Reprogramming by miR-302/367 cluster has shown tumor suppressive effects on different cancer cells, such as melanoma and colon cancer cells (14), cervical carcinoma cells (15) glioblastoma cells (16), prostate cancer cells (13), endometrial cancer cells (17) and breast cancer (18). The miR-302/367 cluster has been shown to induce reprogramming of somatic cells through multiple pathways, including MECP1/2 and AOF1/2 silencing, repression of suppressor NR2F2 gene expression, and silencing RHOC and TGFBRII (19). 

Transforming growth factor-b (TGF-β) signaling pathway is one of the major players in malignant progression through multiple mechanisms which enhance tumor cell invasion, dissemination, and immune evasion (20, 21). In this study we aimed to investigate how overexpression of miR-302/367 cluster in breast cancer cells affects some of the main TGF-β signaling pathway mediators. 

## Materials and Methods

### Cell lines and culture conditions

In this experimental study, human MDA-MB-231 and SK BR-3 breast cancer cell lines were respectively purchased from Pasteur Institute and Iranian Biological Resource Center (IRBC), Iran. Both cell lines were cultured in Dulbecco’s Modified Eagle’s medium (DMEM) with 10% fetal bovine serum (FBS), 1% L-glutamine and 1% penicillin-streptomycin (all from Gibco^TM^, Thermo Fisher Scientific, USA) at 5% CO_2_ and 37°C. The culture medium was renewed every other day.

### Transfection with miR-302/367 expressing vector 

Transfection of MDA-MB-231 and SK-BR-3 were performed using either a TDH101PA-GP miR-302abcd/367 expressing Lentivector (System Biosciences, SBI, USA) or the same vector without the miR-302/367 cluster as the mock control type, using Lipofectamine^®^ 2000 transfection reagent (Invitrogen, Thermo Fisher Scientific, USA) according to the manufacture’s protocol. 48 hours after transfection, transfected cells were selected by adding 1 mg/ ml puromycin dihydrochloride (Bio Basic Inc., Canada) to the culture medium every other day up to the elimination of untransfected cells. Transfected cells were kept in culture condition for a two-week period. 

### Analysis of miRNA and gene expression by quantitative real time polymerase chain reaction

For analysis of miRNA expression, total RNA including small RNA, was extracted from the cultured cells using RNX-Plus solution (Sinaclon, Iran) according to the manufacturer’s protocol. Equal amounts of RNA were reverse transcribed into cDNA using BON-miR miRNA 1st-Strand cDNA Synthesis Kit (Stem Cell Technology Co., Iran). 

For quantification of mRNAs, total RNA was extracted using the High Pure RNA Isolation Kit (Roche, Germany) according to the manufacturer’s protocol. RNAquality and quantity were assessed using a NanoDrop^TM^ 2000/2000c Spectrophotometer (Thermo Fisher Scientific, USA). Equal amount of total RNA from each group was reverse transcribed into cDNA using oligo-dT primers and RevertAid H Minus Reverse Transcriptase (Thermo Fisher Scientific). Assessment of miRNA and mRNA expression was performed, using FastStart SYBR Green Master (Roche, Germany) and specific primers for *miR302a, miR-302b, miR-302c, miR-302d, miR-367* and other genes as mentioned in Table 1, on a Rotor-Gene 6000 (Corbett Research, Australia) real-time PCR instrument. *SNORD47* was selected as the internal reference gene for quantification of miRNAs. *GAPDH* and *B2M* were used as the internal reference genes for quantification of the mRNAs. Comparative analysis of gene expression between different groups was performed using REST 2009 software (Relative Expression Software Tool, Qiagen) based on a Pair Wise Reallocation Randomization Test (22). Four replicates of each group were included in the qPCR reactions. 

**Table 1:** Primers used for quantitative real-time polymerase chain reaction


Target	Primer sequence (5ˊ-3ˊ)	Size (bp)	Accession no.

TGFBR2	F: CCCATCCACTGAGACATATTAAT	198	NM_001024847.2
	R: CATTCTTTCTCCATACAGCCAC		
BUB1	F: GAGTCAAATATGGAACGAAGAG	207	NM_004336.4
	R: GTCTTCATTTACCCATTGCTCA		
RHOC	F: CCTGACAGCCTGGAAAACAT	153	NM_175744.4
	R: AACGGGCTCCTGCTTCATCT		
AKT1	F: ACAAACGAGGGGAGTACATCAA	156	NM_005163.2
	R: TCTTCATCAGCTGGCACTGC		
MAPK1	F: ATTCCAAGGGCTACACCAAGT	136	NM_002745.4
	R: GGATCCAAGAATACCCAAAATGT		
MAPK14	F: TGGCTGTCGACTTGCTGGA	189	NM_001315.2
	R: CATAGGTCAGGCTTTTCCACT		
SMAD3	F: CATAATAACTTGGACCTGCAGC	236	NM_005902.3
	R: ACGCCTCTTCCGATGTGTCT		
B2M	F: TCCAGCGTACTCCAAAGATTCA	113	NM_004048.2
	R: GTCAACTTCAATGTCGGATGGAT		
GAPDH	F: TCACCATCTTCCAGGAGCGA	116	NM_002046.5
	R: CAAATGAGCCCCAGCCTTCT		


### Analysis of cell cycle by flow cytometry

At the end of transfection and cell culture period, the cells were harvested and fixed in 70% cold ethanol and DNA content was stained with propidium iodide (PI) solution. Four replicates of each group were used in this study. Cell cycle analysis was carried out using a FACSCaliburTM flow cytometer (BD Biosciences, USA). FlowJo vX.0.7 software (Tree Star Inc., USA) was used for analysis of the results. Comparison of the cell cycle G1, S and G2/M proportions was performed between the mock and miR-302/367 transfected group of each cell line, using unpaired t test. 

## Results

### Overexpression of the miR-302/367 members in transfected cells 

**Fig.1:**
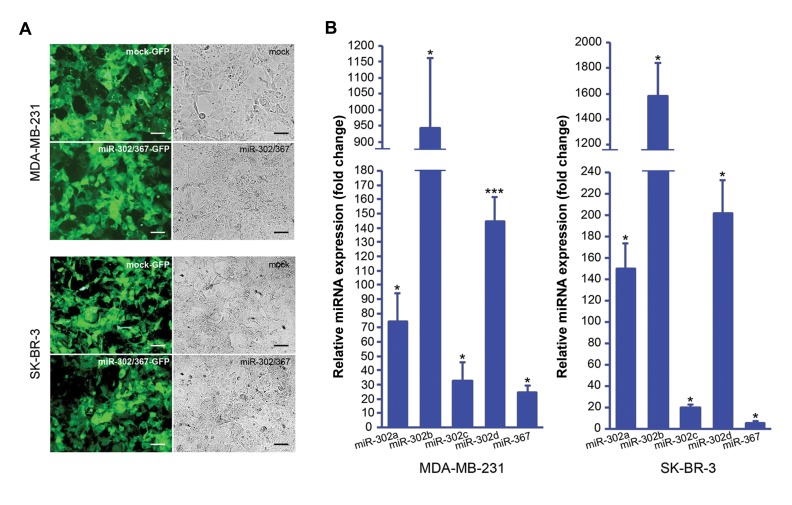
Ectopic expression of miR-302 cluster in the BC cells. **A.** Photomicrographs of the MDA-MB-231 and SK-BR-3 cells transfected with either miR-302/367 or mock vector. Transfected cells show GFP expression. Scale bar represents 50 µm and **B.** Assessment of miR-302/367 expression using quantitative real-time polymerase chain reaction (qPCR) in MDA-MB-231 cells (left) and SK-BR-3 cells (right) transfected with miR-302/367 vector. Fold changes are reflected on the vertical axis compared to the control group (transfected with mock vector) which has been normalized to 1. Analysis performed by REST 2009 software based on a Pair Wise Fixed Reallocation Randomisation Test. and significant P values (*; P<0.05, ***; P<0.001) are indicated on the chart. BC; Breast cancer and GFP; Green fluorescent protein.

Antibiotic-based selection of the miR-302/367 transfected breast cancer cells caused producing a highly (>90%) GFP-expressing cells population (Fig .1A) which were used for the subsequent experiments. Quantification of the *miR-302/367* expression in MDA-MB-231 cells showed upregulation of *miR-302a, miR-302b, miR-302c, miR-302d* and *miR-367* by mean factors of 74, 946, 33, 145 and 25, respectively (Fig .1B). In SK-BR-3 cells, after miR-302/367 transfection, *miR-302a, miR-302b, miR-302c, miR-302d* and *miR-367* were upregulated by mean factors of 145, 1581, 20, 202 and 6, respectively (Fig .1B). 

### Regulation of TGF-ß and MAPK pathway genes by miR-302/367 cluster

**Fig.2:**
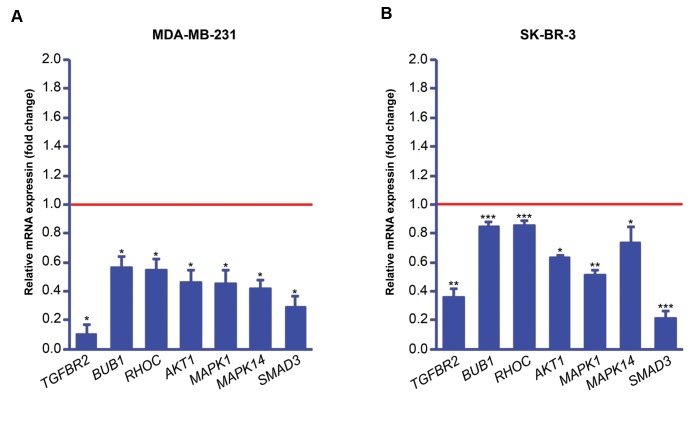
Expression analysis of some transforming growth factor-beta (TGF-β) mediators at mRNA level after transfection with miR-302/367 vector using quantitative real-time polymerase chain reaction (qPCR). Downregulation of TGF-β-related genes in **A.** MDA-MB-231 and **B.** SK-BR-3 cells. Red line represents expression level in the mock transfected group. P values were generated by REST 2009 software based on a Pair Wise Fixed Reallocation Randomisation Test.. Significant P values (*; P<0.05, **; P<0.01, ***; P<0.001) are reflected on the chart.

Firstly, we checked how transfection of the breast cancer cells with miR-302/367 cluster affects the expression of some key mediators of TGF-β and mitogenactivated protein kinase (MAPK) pathways at gene level. Quantitative real-time PCR showed that in the both MDA-MB-231 and SK-BR-3 cells, overexpression of *miR-302/367* cluster downregulated *TGFBR2, BUB1, RHOC, AKT1, MAPK1, MAPK14* and *SMAD3* expression compared to the mock transfected cells (Fig .2). 

### Cell cycle arrest by overexpression of miR-302/367 cluster 

**Fig.3:**
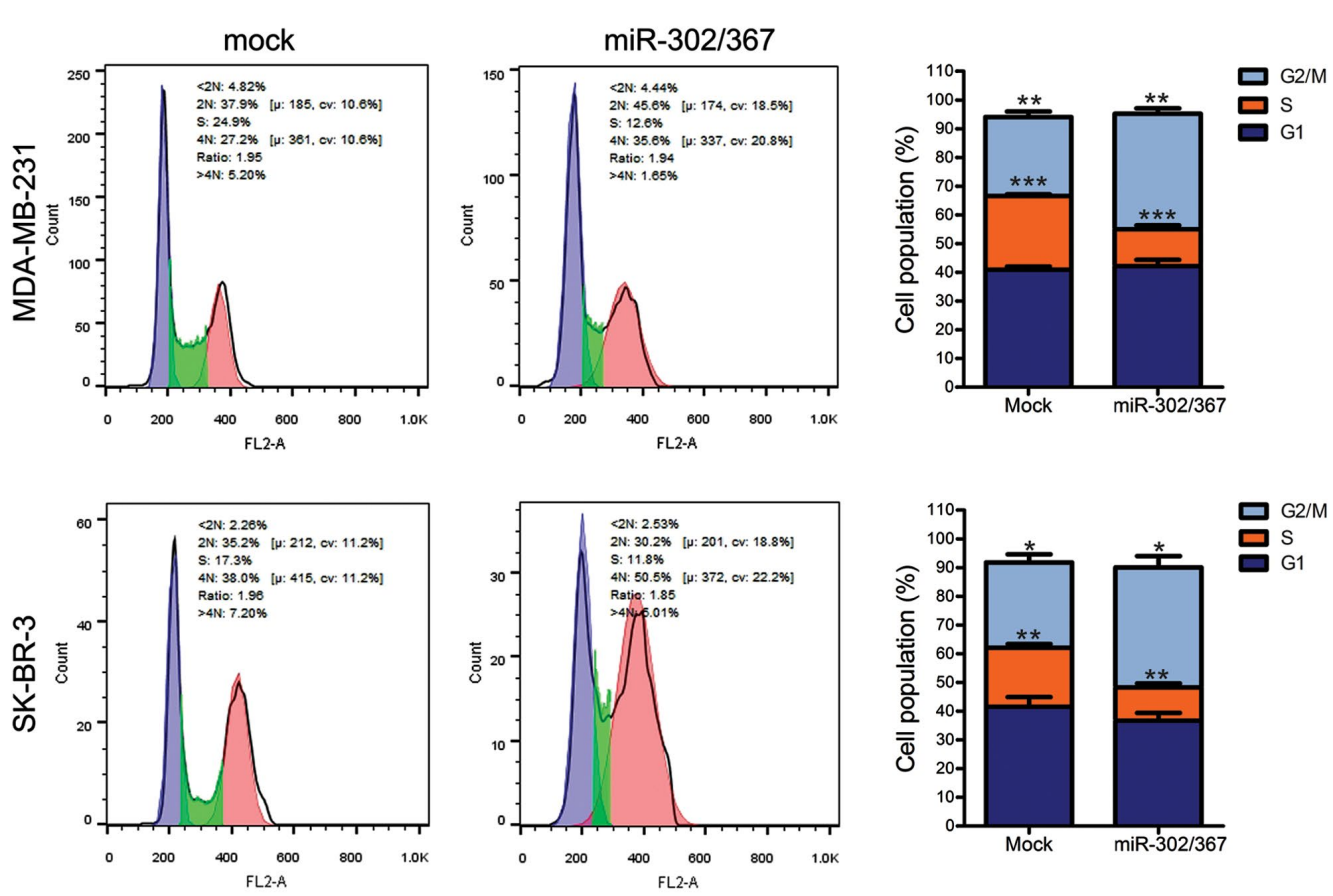
Flow cytometry analysis of cell cycle. There was a significant decrease in S-phase and a partial increase in G2/M-phase population of both MDA-MB-231 and SK-BR-3 cells, after overexpression of miR-302/367 cluster (unpaired t test, n=4, *; P<0.05, **; P<0.01 and ***; P<0.001).

At the end of culture period, transfected breast cancer cells with either miR-302/367 or mock vector were analyzed for the cell cycle phases, using PI staining and flow cytometry. In the miR-302/367 transfected MDA-MB-231 and SK-BR-3 cells, there was a marked decrease in the S-phase population, while the G2/M phase population was partially increased compared to the mock transfected group (Fig .3). 

**Fig.4:**
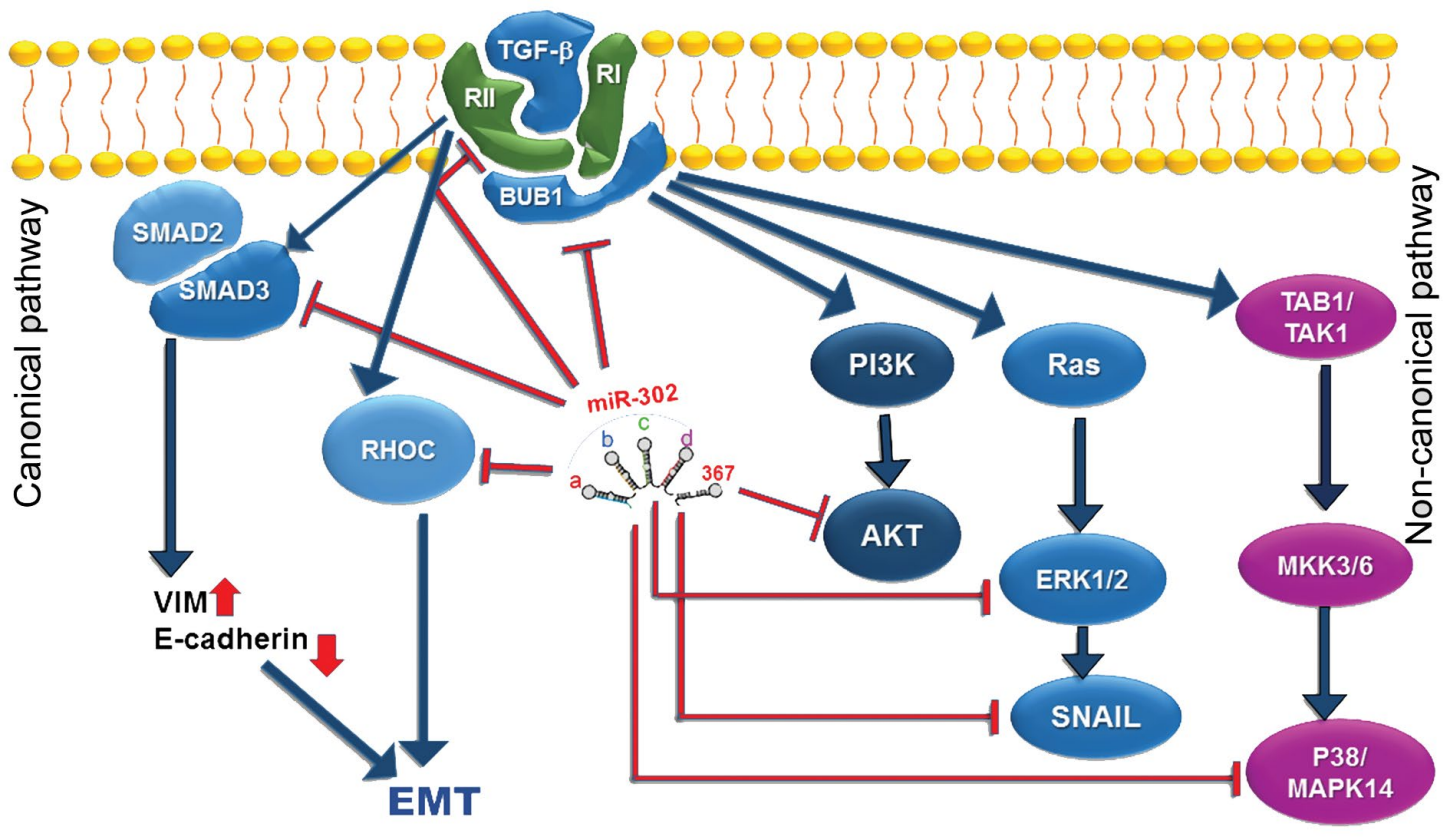
Interaction between miR-302/367 cluster and several mediators of transforming growth factor-beta (TGF-β) signaling in both canonical and non-canonical pathways. The inhibitory effect of miR-302/367 cluster is shown by the red lines.

## Discussion

Genetic and epigenetic alterations contribute to cancer initiation and progression through affecting gene expression. While genetic mutations may lead to stable and irreversible alterations, transient and reversible changes are usually caused by epigenetic modifications (23). It has been shown that reprogramming of cancer cells by some pluripotency transcription factors or specific miRNAs, like miR-302/367 cluster, may lead to an embryonic stem cell-like state and less tumorigenicity (13, 24). We previously demonstrated upregulation of some pluripotency factors, including OCT4A, SOX2 and NANOG, by overexpression of *miR-302/367* cluster in MDA-MB-231 and SK-BR-3 cells (18). 

Accumulating evidence supports the function of miR 302 cluster as a tumor suppressor family which can alleviate tumorigenicity of cancer cells through reversal of epithelial to mesenchymal transition (EMT), induction of apoptosis and anti-proliferative effect (13-15). Previously, we demonstrated some anti-tumor effects of miR-302 cluster in melanoma, colon and breast cancer cells (14, 18). Among the pathways promoting EMT, several studies have reported that inhibition of TGF-ß signaling induces somatic cell reprogramming (25, 26).

In this study, we investigated how overexpression of *miR-302/367* cluster in MDA-MB-231 and SKBR- 3 breast cancer cells affects some mediators of TGF-β/MAPK/AKT signaling pathway at gene level. As shown, *TGFBR2* and *RHOC* are directly targeted by miR-302 cluster, subsequently facilitating human or mouse fibroblast reprogramming towards iPS cells (27, 28). In accordance with these studies, we found that overexpression of *miR-302/367* in human breast cancer cells downregulates *TGFBR2* and *RHOC* expressions. TGF-ß signaling has two canonical and non-canonical pathways (Fig .4). In the canonical or SMAD-dependent pathway, SMAD proteins play key regulatory roles among which SMAD2 and SMAD3 proteins are phosphorylated through activity of TGF-ß and activin (29). While, in the non-canonical or SMAD-independent pathway, TGF-ß activates phosphatidylinositol 3kinase (PI3K)/ AKT and MAPK pathways (30). In the current study, expressions of *TGFBR2, BUB1, RHOC, AKT1, MAPK1, MAPK14* and *SMAD3* were significantly downregulated after overexpression of *miR-302/367* cluster in the both cell lines. Previously, Cai et al. (15) reported that miR-302/367 directly targets *AKT1* and it suppresses proliferation of HeLa and SiHa cervical carcinoma cells. In the same study, AKT1 protein level was decreased after miR-302/367 transfection, but *AKT1* gene expression was not significantly changed. In another study, Li et al. (31) demonstrated that miR-302abcd cluster upregulated OCT4 expression by targeting *AKT1* gene at its 3’-UTR. The same report also showed downregulation of AKT1 at both gene and protein levels, following miR-302 transfection. Similarly, we showed that overexpression of *miR-302/367* cluster in MDA-MB-231 and SKBR- 3 cells induces expression of *OCT4* gene (18) and downregulates expression of *AKT1*. Therefore, it seems that a mechanism, similar to that of previous reports, is applicable to breast cancer cells. 

We also detected a significant downregulation of *SMAD3* expression following miR-302/367 transfection of the breast cancer cells. Sustained activity of SMAD complexes in the nucleus is one of the key features of TGF-ß signaling in cancer cells. It was reported that an interaction between FOXM1 and SMAD3 is critical for TGF-β-mediated gene expression. Thus, it promotes breast cancer cell invasion and metastasis (32). 

*BUB1* was also downregulated in the breast cancer cells after ectopic expression of *miR-302/367* cluster. BUB1 is a serine/threonine kinase playing a significant role in cell cycle regulation, chromosome cohesion (33), and it is a key mediator of TGF-β signaling. It has been shown that BUB1 promotes canonical and non-canonical TGF-β signaling and mediates TGF-β-dependent EMT, cell migration and invasion through interaction with both TGFBRI and TGFBRII (34). Here, for the first time, we are reporting downregulation of *BUB1* expression in breast cancer cells following overexpression of *miR302/ 367* cluster. This provides further evidence regarding the significance of miR-302/367 suppressive impact on TGF-β signaling through inhibition of both canonical and non-canonical pathways. 

MAPK pathway is part of the non-SMAD pathways, activated by the TGF-ß receptors (35). MAPK1, also known as ERK2, is encoded by *MAPK1* gene. The ERK1/2 pathway plays a pivotal role in regulation of cell proliferation, and it is known as a master regulator of G1 to S-phase progression (36, 37). Another player of the non- canonical TGF-β pathway, MAPK14/p38α is encoded by *MAPK14* gene. MAPK14/p38α is 50% identical to ERK2 and generally expressed in cell lines and tissues (36). There has been controversy regarding the role of p38 MAPKs in regulating cell proliferation and survival (38). This primarily depends on the cell type determining whether p38 MAPK induces progression or inhibition of G1/S transition through differential regulation of cyclin A or D levels, phosphorylation of RB protein (39, 40), and phosphorylation of p53 (40). In our study, ectopic expression of *miR-302/367* cluster in the breast cancer cells downregulated expression of all of the investigated TGF-β mediators, including *TGFBR2, BUB1, RHOC, AKT1, MAPK1, MAPK14* and *SMAD3*. These findings indicate a strong suppressive effect of miR-302/367 cluster on the TGF-β signaling (Fig .4). In this study we report a lower proliferation rate and S-phase suppression of the breast cancer cells by overexpression of *miR-302/367,* confirming our previous report (18). This can be explained by suppression of MAPK1 and MAPK14 to some extent. Therefore, suppression of TGF-β mediators may provide a good reason behind the partial cell cycle arrest observedin the breast cancers, following overexpression of *miR302/ 367* cluster. 

## Conclusion

Overexpression of *miR-302/367* cluster in human breast cancer cells resulted in a general suppressive effect on multiple mediators of TGF-β signaling and BUB1. This finding was accompanied by inhibition of cell proliferation. Previously, we reported anti-tumor Ahmadalizadeh Khanehsar et al. effects of either miR-302bcad or miR-302bcad/367 clusters on melanoma, colon and breast cancer cells due to induction of apoptosis and suppression of proliferation and invasion. Current results are providing new evidence that suppression of TGF-β signaling at gene level may be one of the important reasons for anti-tumor effects of miR-302/367 cluster in breast cancer cells. 
